# Twenty years of *PLOS One*

**DOI:** 10.1371/journal.pone.0345574

**Published:** 2026-04-03

**Authors:** Emily J. Chenette

**Affiliations:** PLOS, Cambridge, United Kingdom; Public Library of Science, UNITED KINGDOM OF GREAT BRITAIN AND NORTHERN IRELAND

PLOS started in 2000 with an open letter written by Harold Varmus, Patrick Brown and Michael Eisen [1]. This letter urged publishers to make research literature available to all through free online public archives:

*We support the establishment of an online public library that would provide the full contents of the published record of research and scholarly discourse in medicine and the life sciences in a freely accessible, fully searchable, interlinked form. Establishment of this public library would vastly increase the accessibility and utility of the scientific literature, enhance scientific productivity, and catalyze integration of the disparate communities of knowledge and ideas in biomedical sciences.*

Nearly 34,000 scientists from 180 countries signed the letter.

Despite the international support for a Public Library of Science, little changed until PLOS (a non-profit since inception) became an open access publisher. Its mission was straightforward: to provide a mechanism to disseminate research so that readers anywhere could access trustworthy information at no cost. PLOS launched *PLOS Biology* in 2003, followed by *PLOS Medicine* in 2004, and *PLOS Computational Biology*, *PLOS Genetics* and *PLOS Pathogens* in 2005.

## 2006: The launch of *PLOS One*

The success of these first PLOS journals demonstrated that the global research community strongly supported PLOS’ mission. However, PLOS saw the need for a different kind of journal: a multidisciplinary, open access journal with publication criteria that prioritized ethical and technical rigor, but that left the determination of the paper’s “impact” to readers *after* publication, not by editors and reviewers before publication. With these guiding principles in mind, *PLOS One* opened for submissions in August 2006, and the first research articles were published in December of that year.

**Fig 1:**
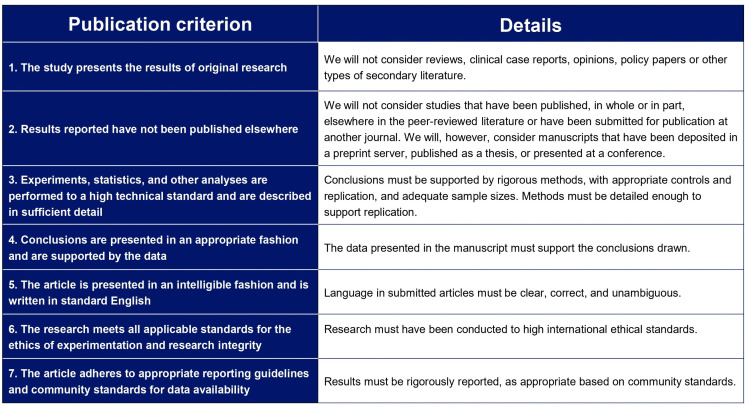
*PLOS One’s* seven criteria for publication.

Manuscripts submitted to *PLOS One* were accepted for publication if they met all seven publication criteria (Fig 1).

To ensure that all publications met these criteria and adhered to PLOS’ policies, we introduced an innovative submission scan (“subscan”) check, which was performed on all new submissions before they were sent to an Academic Editor for peer review. Subscan is performed by subject matter experts with advanced degrees in a relevant subject area, and focuses on adherence to PLOS policies. Academic Editors and reviewers could then focus solely on the scientific content of the manuscript.

## 2007-2013: Navigating the challenges of publishing at scale

The community response to the launch of *PLOS One* was far greater than expected. In the first 7 years after launch, publications in *PLOS One* grew significantly year to year. But with that growth came significant challenges.

As a first step towards addressing these challenges, we implemented additional editorial policies to guide decision-making. Changes to our policies were discussed extensively with members of our Editorial Board, as well as other researchers, to ensure they represented community-endorsed and field-appropriate practices. For example, in 2012, we developed a policy to increase reproducibility, accessibility and ethical standards in paleontology and archaeology research [2], and we released specific criteria for manuscripts reporting the development of methods, software, databases and tools [3]. In subsequent years, we updated our animal ethics policies to provide additional guidance around humane endpoints, anesthesia and euthanasia [4], and released new guidelines for meta-analyses of genetic association studies [5].

We then turned our attention to the *PLOS One* Editorial Board. To ensure the board had sufficient capacity to handle the submissions we received we created a department within PLOS that is responsible for recruitment, training and engagement of Editorial Board members. This department enforces strict vetting criteria for new board members, regularly assesses board composition and activity, and manages a feedback and offboarding pathway for Academic Editors who do not uphold our Code of Conduct. As a result, the *PLOS One* Editorial Board contains active researchers who are best placed to make decisions around methodology, discussion of results, and appropriateness of conclusions. Communicating clear standards for their work as Academic Editors, and enforcing those standards, helps to safeguard the integrity of what we publish.

Finally, we expanded the in-house editorial team, and optimized publishing workflows. Increasing the number of highly trained staff editors, who work in close consultation with reviewers and Academic Editors, ensured that all submissions to *PLOS One* had a path to resolution with in-house decisions made for the small subset of manuscripts that might otherwise face delays. This was coupled with limited outsourcing and the development of additional workflows that also reduced delays. These might all sound like obvious steps now, but back in 2013, no journal had experienced the volume of submissions that came to *PLOS One*.

## 2014-2020: Pioneering open science practices

This explosive growth at *PLOS One* led others to take notice. Soon, other publishers began launching new open access mega-journals. PLOS welcomed these new journals as they represented an exciting step towards realizing our original mission of increasing adoption of open access publishing across the research ecosystem [6]. However, even though other journals launched on the same inclusive model, *PLOS One’s* focus on open science, ethics and integrity set it apart.

**Fig 2:**
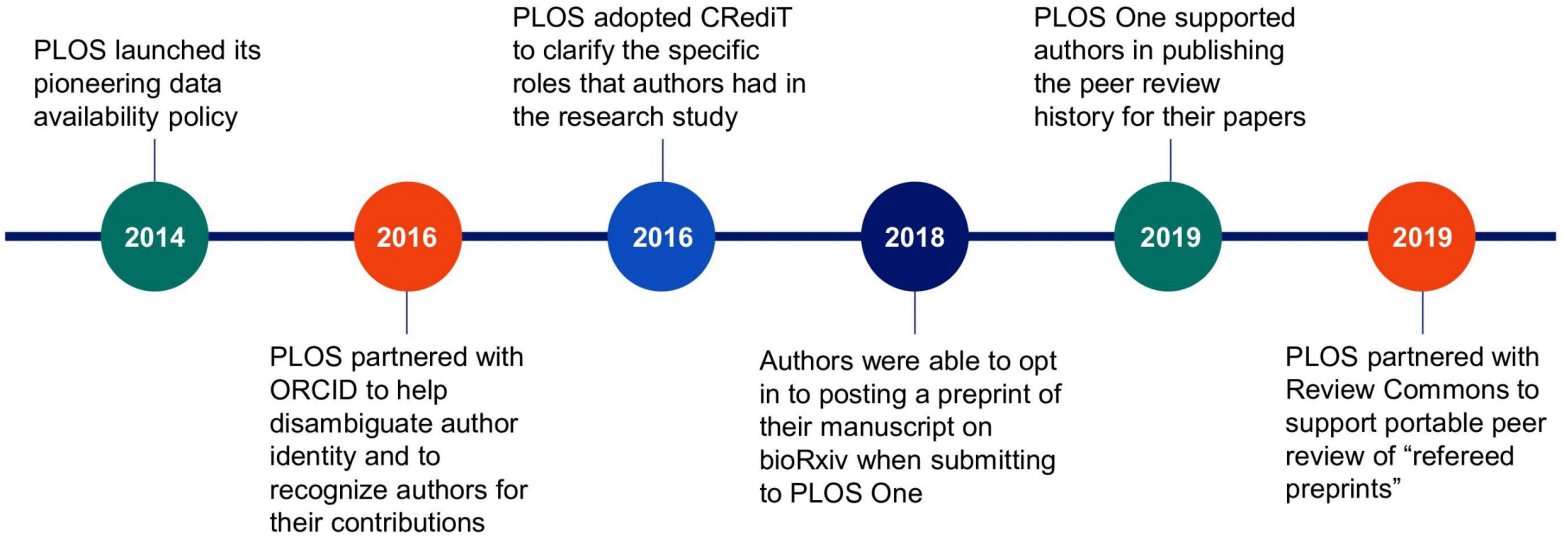
Key milestones in PLOS’ journey to advance open science via its research journals.

With additional publishing options now available, the output of *PLOS One* peaked in 2013, decreasing gradually over the next several years. At the same time, research culture was evolving, and we began to pioneer new ways to increase the adoption of open science practices. Key milestones in this journey are shown in Fig 2.

## 2021-2026: Threats and opportunities in a shifting publishing landscape

The past 5 years have brought new threats and opportunities to academic publishers: a surge of COVID-related submissions, the rise of AI, and an increase in suspicious submission activity. At *PLOS One*, these challenges led to the development of additional industry-leading checks and policies aimed at disrupting potentially troubling trends.

For example, in 2022 we announced a policy on inclusivity in global research [7] to address concerns around the increasing problem of “parachute” or “helicopter” research. Developed in close collaboration with researchers around the world, the policy requires authors to complete a checklist that aims to improve transparency in the reporting of research performed outside of a researcher’s own country or community. The policy helps ensure that PLOS publications reporting global research adhere to high standards for research ethics and authorship.

In 2023, we announced a new policy for submissions reporting research involving human participants or their data [8]. *PLOS One* has always required that such submissions include a statement about the study’s ethics approval and informed consent procedures. The 2023 update required authors to provide their ethics approval documentation and led to a 30% increase in rejections at initial submission.

We continue to develop new policies to address emerging threats to the integrity of academic publishing. Our proactive monitoring of submission trends spotted a spike in submissions of systematic review and meta-analyses: they increased 27% from 2022–2023, and another 33% from 2023–2024. In late 2024, after consultation with Editorial Board Members and researchers with specific expertise in systematic reviews and meta-analyses, we introduced new checks on these study types to ensure that they complied with best practice in research reporting. In the past year, we’ve also introduced new checks on Mendelian randomization studies [9] as well as studies involving open health databases [10].

Most recently, in 2025, PLOS introduced a comprehensive AI policy outlining acceptable use (language polishing) and unacceptable use (fabrication or misrepresentation of text or figures) of this technology [11]. All AI use must be disclosed in the manuscript, and authors are responsible for ensuring that content is valid, all relevant sources are cited, and that all statements and text represent the authors’ own ideas. Together, these policies have ensured that the growth in *PLOS One* in recent years has been driven by reproducible, rigorous research.

## Lessons learned

Over the past 20 years, we learned that there is broad community support for *PLOS One’s* founding principles. Before *PLOS One* launched, no one knew if a multidisciplinary, inclusive journal focused on rigor, reproducibility and transparency would be successful. However, the sustained high submission volume to *PLOS One* and other open-access mega-journals shows widespread and lasting support for this publishing model.

The most enduring lesson we have learned is that our principles matter more than profits. The new policies that we have introduced to address changing threats to research integrity have increased rejection rates. However, we remain committed to our founding principles of ensuring that *PLOS One* publishes only ethically and technically rigorous research, free for readers around the world to learn from, reuse and build upon to advance scientific knowledge.

## References

[1] Open Letter. Accessed 2026 February 25. https://plos.org/open-letter/

[2] Paleontology and Archaeology Research. Accessed 2026 February 25. https://journals.plos.org/plosone/s/animal-research#loc-paleontology-and-archaeology-research

[3] Materials, software and code sharing. Accessed 2026 February 25. https://journals.plos.org/plosone/s/materials-software-and-code-sharing

[4] Animal Research.Accessed 2026 February 25. https://journals.plos.org/plosone/s/animal-research

[5] Meta-analysis of genetic association studies. Accessed 2026 February 25. https://journals.plos.org/plosone/s/submission-guidelines#loc-meta-analysis-of-genetic-association-studies

[6] Allen L. Welcome, nature. Seriously. PLOS Blogs. 2011. Accessed 2026 February 25. https://theplosblog.plos.org/2011/01/welcome-nature-seriously-2/

[7] Announcing a new PLOS policy on inclusion in global research. 2021. Accessed 2026 February 25. https://theplosblog.plos.org/2021/09/announcing-a-new-plos-policy-on-inclusion-in-global-research/

[8] Hoch R, Chenette EJ. The value of enhanced ethics checks: Initial experiences with PLOS ONE’s updated Human Subjects Research policy. PLoS One. 2023;18(8):e0288900. doi: 10.1371/journal.pone.0288900 37556432 PMC10411757

[9] Pruller J, Mockridge J, Vousden G. Expectations for Mendelian randomization research in PLOS One. PLoS One. 2025;20(11):e0337199. doi: 10.1371/journal.pone.0337199 41252429 PMC12626271

[10] Updates to PLOS retrospective health database editorial policy. 2025. Accessed 2026 February 25. https://theplosblog.plos.org/2025/09/updates-to-plos-retrospective-health-database-editorial-policy/

[11] Artificial Intelligence Tools and Technologies. Accessed 2026 February 25. https://journals.plos.org/plosone/s/ethical-publishing-practice#loc-artificial-intelligence-tools-and-technologies

